# Good Health

**Published:** 1891-07

**Authors:** 


### HALL'S JOURNAL OF HEALTH

TRUTH DEMANDS NO SACRIFICE; ERROR CAN MAKE NONE.

Vol. 38. · JULY, 1891. No. 7.

### GOOD HEALTH.

Good health does not always come to our door. It is not carried about and delivered upon order, by the grocer, the baker and the ice-man. We are oftentimes compelled to seek it away from home, in outdoor rambles, in field, in forest, or by the ever changing sea. In these midsummer days, Nature in her loveliest attire offers us the rarest enticements to partake of her bounty.

> "There is a pleasure in the pathless woods,
> There is a rapture on the lonely shore;
> There is society where none intrudes,
> By the deep sea, and music in its roar."

It is not alone the body, but the mind also needs to be diverted and turned into new channels of thought and action. This is not only true of invalids, but those who are in daily attendance upon business pursuits of their own choosing, require intervals of relaxation, wherein to recuperate their impaired vitality. The necessity of this is so generally conceded that the summer vacation is looked forward to almost as a matter of course, in all trades and employments; and we affirm out of our own experience, that it is no less a necessity than a pastime. The homely couplet, "All work and no play makes Jack a dull boy," is a truism which should not be lost sight of.

Few people appreciate properly the hygienic powers of sunlight. It is true of people, as it is true of plants, that they cannot thrive without abundance of sunlight, as well as abundance of fresh air. The necessity for sunlight is so well recognized that in all the recent lectures to nurses of the sick, they are ordered to admit the sunshine freely to the sick room in all cases, except where the strong light is specially prohibited by the physician.

Not long ago sun baths were freely recommended for certain diseases, and this treatment has since proved exceedingly valuable, so much so, indeed, that complete systems of treatment and cure, with sunshine as a basis, are much in vogue in private practice and sanitary institutions.

The Orientals, who have gardens on the tops of their houses, appreciate the value of sunlight as a tonic and health giver. The cases of persons who suffer from actual sunstroke are much fewer than of those who suffer unto death from vitiated air and want of sunshine. The mass of cases reported as sunstroke in the cities are the result of prostration from heat, and occur in close rooms within doors as frequently as outdoors. In most of these cases the deteriorated condition of the system of the individual, caused by the confinement in rooms insufficiently aired and lighted, is at the bottom of the trouble.

It is especially necessary that children should have an abundance of freedom to romp outdoors in the sunshine, so that they will acquire an abundance of red blood, and with it strength and life. Pale, sallow complexions show a watery condition of the blood that can only be remedied by an abundance of outdoor exercise. In winter, it is always best to give a little child its exercise in the middle of the day ; but as the season changes the time for exercise changes. In summer, the best time is usually early in the morning, before 10 o'clock, and after 3 in the afternoon. In the morning, a rubber sheet should, if the ground is damp, be spread in a suitable place over the grass and a blanket spread over this, and the little one taken out of his carriage and allowed to frolic about in the mild morning sun. The baby will gain marvelously from such exercise, and it will be all the better off if it is kept under the trees to take its midday nap, instead of being taken into the house.

The invention of an outdoors game, in which both sexes are wont to participate, is a public benefaction.

Croquet and Lawn Tennis have cheated the family doctor of many a professional visit, and will continue to do so, it is to be hoped, indefinitely. All the organs of the body require to be continually exercised. They cannot exist without it. In a child, before it is restricted by the tyranny of fashion, every movement is grace itself. It should be so always, but the satanic invention of high heeled shoes, the straight-jacket of a corset, together with old maidish notions of propriety, are at the foundation of many physical ills.

